# Widely Linear Adaptive Instantaneous Frequency Estimation in Vector Hydrophones

**DOI:** 10.3390/s18103348

**Published:** 2018-10-07

**Authors:** Panpan Peng, Liang An

**Affiliations:** Key Laboratory on Underwater Acoustic Signal Processing of MOE, Southeast University, Nanjing 210096, China; 220160753@seu.edu.cn

**Keywords:** widely linear, frequency estimation, ACLMS, vector hydrophone

## Abstract

To solve the problem that the time-frequency resolution of Short-Time Fourier Transform (STFT) is constrained by the window length and the moving step of the short time window, and to utilize the merits of a widely linear method, a novel instantaneous frequency estimation method in vector hydrophone was proposed. In this paper, a complex variable was constructed. It is composed of sound pressure and particle velocity as its real part and imaginary part, respectively. The constructed variable was approved to be second order noncircular (improper). For the modelling of noncircular signals, the standard linear estimation is not adequate and the pseudo-covariance matrix should also be taken into consideration. As a result, a widely linear adaptive instantaneous frequency estimation algorithm and its three solutions based on the augmented complex least mean square (ACLMS) method are presented to estimate the instantaneous frequency in vector hydrophones. The results of simulations and laboratory experiments prove that this approach based on a widely linear model performs better compared to STFT and strict linear filter methods.

## 1. Introduction

Instantaneous frequency estimation is an important issue in underwater acoustic signal processing. The most widely adopted method used to estimate the frequency in underwater acoustics systems is the Short-Time Fourier Transform (STFT), which is a traditional instantaneous frequency estimation method widely used in the time-frequency analysis field because of its simplicity. Another advantage of STFT is that it can reflect the time-frequency information of the signal accurately without the interference of cross terms. However, like most time-frequency methods, there are two limits. The time-frequency resolution is constrained by the window length and the moving step of the short time window and besides, its anti-noise performance is poor [1]. As a result, STFT is not appropriate for analysis of non-stationary signals or signals with fast-changing frequency like LFM signals.

As an alternative to the traditional Fourier Transform, Xia et al. [2,3,4] proposed the widely linear model for frequency estimation in three-phase power systems. It is illustrated that the complex-valued signal, obtained by the αβ transformation of three-phase power signals under unbalanced voltage sag conditions is second order noncircular, for which standard linear adaptive estimators are suboptimal. The proposed algorithm combines the merits of the widely linear model and adaptive filter based on the minimization of the mean square error. This approach offers enhanced accuracy and faster convergence, together with robustness to noise. Similar widely linear filtering methods are applied in acoustic echo cancellation [5,6]. Paleologu et al. [5] proposed widely linear Kalman filter method that combines the advantages of the widely linear model and the good features of the Kalman filter, and outperforms the recursive least square method. Huang et al. [7] and Xu et al. [8] presented widely linear minimum dispersion-based beamforming for sub-Gaussian noncircular signals, taking full advantages of the non-circularity and sub-Gaussian properties of signals. The approach in [7,8] is shown to achieve good performance, even in high signal-to-noise ratio conditions and is able to process more signals than the number of sensors.

Based on the merits of applying widely linear filtering in some fields, it seems that combining widely linear filtering with vector hydrophones is meaningful, since vector hydrophones play an increasingly important role in submarine resources exploration and marine military activities. Compared with scalar hydrophones which can only measure sound pressure, vector hydrophones have attracted much more attention due to their characteristics of good directivity [9,10,11,12]. Acoustic vector sensors measure scalar acoustic pressures along with particle velocity in three orthogonal directions and find a wide range of applications in underwater acoustics tasks such as coastal surveillance, harbor defense, underwater acoustic communication, and source localization studies [13,14,15,16]. Theoretically, the sound pressure and particle velocity have a phase difference of 90°, which provides the possibility that a noncircular complex signal can be constructed and it can be used for widely linear frequency estimation based on the most recent advances in augmented complex-valued second order statistics.

In most cases, a widely linear model is superior to a traditional strictly linear model when dealing with second order signals. We propose to use the recently introduced widely linear modelling- based adaptive filtering algorithm, called the Augmented Complex Least Mean Square (ACLMS) to deal with instantaneous frequency estimation in vector hydrophones. This is a novel approach towards frequency estimation in underwater acoustic signal processing, because nearly no one has introduced this method in vector hydrophones before. Firstly, it is illustrated that the constructed complex variable composed of sound pressure and particle velocity as its real part and imaginary part, respectively, is second order noncircular (improper) due to its noncircular rate approaching 1. For the modelling of noncircular signals, the standard linear estimation is not adequate and the pseudo-covariance matrix should also be taken into consideration. Actually, the key point of the advanced performance is to take the signal’s complex conjugate into account. Then, a widely filter model and its three solutions based on the ACLMS method are then presented to estimate the instantaneous frequency of the complex signal, in contrast with what is deduced in paper [2] where another two solutions were ignored. Simulation results and the results of laboratory experiments prove that this widely linear based method performs better compared to STFT and other linear filtering methods.

## 2. Background

### 2.1. Widely Linear Modelling and Basic Characteristics

Consider a real-valued conditional mean squared error estimator (MSE) of a random vector y in terms of a real observation x. For zero-mean jointly normal y and x, the optimal estimator is linear. In the complex domain, it is typically assumed that the same form of estimator can be used leading to the standard complex linear minimum mean square error estimator [2,17]:

(1) $$\hat{y}={\hat{y}}_{r}+j{\hat{y}}_{i}=x^{T}h$$

where $h={\left[h_{1},\cdots ,h_{L}\right]}^{T}$ is a vector of fixed filter coefficients, $x={\left[x_{1},\cdots ,x_{L}\right]}^{T}$ is the regressor vector, and ${\left(\cdot \right)}^{T}$ denotes the vector transpose operator. j is the imaginary unit and subscripts r and i denote respectively the real and imaginary parts of a complex variable. Standard strictly linear estimation in the complex domain assumes the same model but with complex value y, x, h is widely used. Observing that both the real ${\hat{y}}_{r}$ and imaginary ${\hat{y}}_{i}$ parts of the vector y are real-valued, and thus:

(2) $${\hat{y}}_{r}=E\left\{y_{r}|x_{r},x_{i}\right\}\;{\hat{y}}_{i}=E\left\{y_{i}|x_{r},x_{i}\right\}$$

Substituting $x_{r}=(x+x^{*})/2$ and $x_{i}=(x-x^{*})/2j$ yields:

(3) $${\hat{y}}_{r}=E\left\{y_{r}|x,x^{*}\right\}\;{\hat{y}}_{i}=E\left\{y_{i}|x,x^{*}\right\}$$

where ${\left(\cdot \right)}^{*}$ is the complex-conjugate operator, using Equation (1), the widely linear complex estimator for complex valued data is obtained as:

(4) $$\hat{y}=h^{T}x+g^{T}x^{*}$$

where h and g are complex-valued coefficient vectors. Such a widely linear estimator is optimal for the generality of complex signals, and it simplifies into the strictly linear model (g=0) for circular data.

### 2.2. Second Order Noncircular and Circular Signals

The difference between circular and uncircular signals is based on their secondary moment properties [18,19]. For a zero-mean complex random vector s, there exists two definitions of covariance matrix $C_{\mathrm{ss}}=E\left\{SS^{H}\right\}$, $P_{\mathrm{ss}}=E\left\{SS^{T}\right\}$ where $C_{\mathrm{ss}}$ and $P_{\mathrm{ss}}$ are called respectively the covariance matrix and pseudo-covariance matrix, ${\left(.\right)}^{H}$ and ${\left(.\right)}^{T}$ denotes the Hermitian operator and transpose operator, respectively. When $P_{\mathrm{ss}}=0$, the random vector **s** is called circular (proper). Otherwise, it is noncircular (improper). It means that its imaginary and real parts enjoy the same probability density functions and variance. The so-called “proper” data have equal powers in the real and imaginary parts, while for the improper data, the covariance is greater than the absolute pseudo-covariance. In real word, most complex signals are second order noncircular or improper. The advantage of widely linear estimation over strictly linear estimation can be quantified by the difference between the mean square errors of a strictly linear estimator, $e_{L}^{2}$, and that of a widely linear estimator, $e_{WL}^{2}$, given by:

(5) $$\begin{array}{ll} \delta e^{2} & =e_{L}^{2}-e_{WL}^{2}\\ & ={\left[p-P_{ss}^{*}C_{ss}^{*-1}c^{*}\right]}^{H}{\left[C_{ss}-P_{ss}C_{ss}^{*-1}P_{ss}^{*}\right]}^{-1}[p-P_{ss}^{*}C_{ss}^{*-1}c^{*}] \end{array}$$

where $c=E[y^{*}x]$ and p=E[yx]. Because of the positive definiteness of the matrix $\left[C_{ss}-P_{ss}C_{ss}^{*-1}P_{ss}^{*}\right]$, $\delta e^{2}$ is nonnegative.

**Figure 1:**
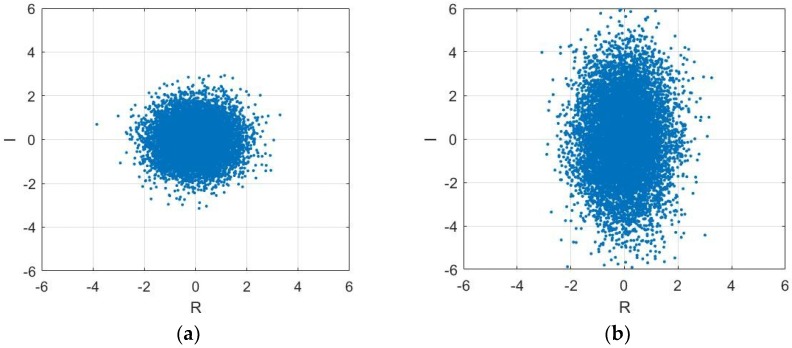
Scatter diagrams of the noncircular signal and circular signals: (**a**) circular signal; (**b**) noncircular signal.

In the simulation process, the noise n(k) is complex white Gaussian noise (WGN) and its real and imaginary parts are independent real WGN sequences, $n(k)=n_{r}(k)+jn_{i}(k)$ and $\sigma_{n}^{2}=\sigma_{nr}^{2}+\sigma_{ni}^{2}$. Figure 1 demonstrates second order circular and noncircular signals visually by setting complex noise with different variances in its real and imaginary parts. Figure 1 shows the simulation results of circular and noncircular signals based on complex-valued AR (4) process. Figure 1a depicts a stable autoregressive AR (4) process driven by doubly white circular Gaussian noise in the form of “real-imaginary” scatter plots in the complex plane. When $\sigma_{nr}^{2}=\sigma_{ni}^{2}$, the circular signal can be seen in Figure 1a and if $\sigma_{nr}^{2}\neq \sigma_{ni}^{2}$, Figure 1b shows the noncircular signal. The degree of non-circularity is used to depict whether a complex signal is circular or non-circular. It can be calculated by using the circularity index:

(6) $$\eta =\frac{\tau_{v}^{2}}{\sigma_{v}^{2}}$$

where $\sigma_{v}^{2}$ is the variance of a complex variable v, and $\tau_{v}^{2}$ is the absolute value of the pseudo-variance of v. This way, η∈[0,1], the value of 0 indicating that v is second order circular, otherwise indicating a second order noncircular signal.

## 3. Frequency Estimation Based on ACLMS in Vector Hydrophone

### 3.1. Deduction of Three Solutions to Frequency Estimation Based on ACLMS

The least mean square (LMS) algorithm is the most usually used stochastic gradient adaptive filtering algorithm which adaptively adjusts the filter coefficients in order to minimize the instantaneous squared error. It is assumed that the complex signal v(k) serves as the desired signal, then ACLMS algorithm can be given by [2,19,20,21]:

(7) $$\begin{array}{ll} \hat{v}(k+1) & =v(k)h(k)+v^{*}(k)g(k)\\ \;\;e(k) & =v(k+1)-\hat{v}(k+1)\\ h(k+1) & =h(k)+ue(k)v^{*}(k)\\ g(k+1) & =g(k)+ue(k)v(k) \end{array}$$

where h(k) and g(k) are respectively the filter weight coefficients corresponding to the standard conjugate parts at time instant k, $\hat{v}(k+1)$ is the estimation of v(k+1), e(k) represents the estimation error and u is the step-size, a convergence factor controlling stability and the rate of adaptation. The algorithm is based on the method of steepest descent.

**Figure 2:**
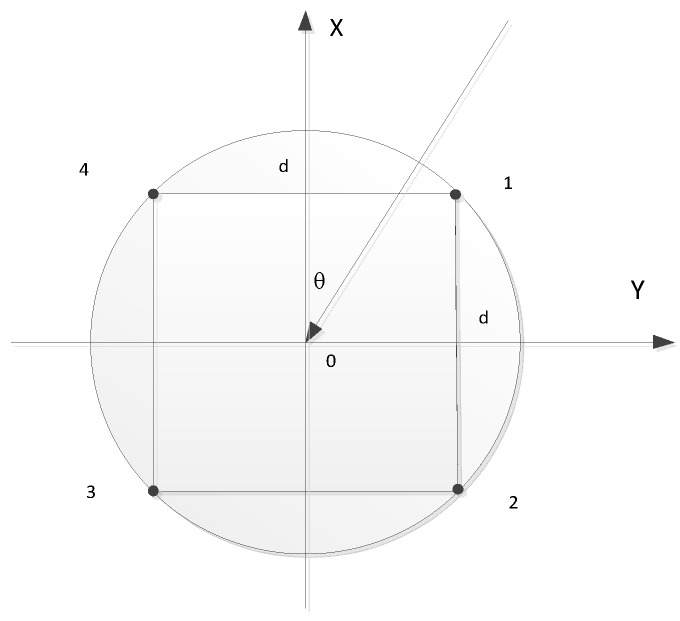
Schematic view of the structure of the pressure-gradient vector hydrophone.

Figure 2 is a schematic diagram of the pressure-gradient vector hydrophone, which is composed of two pairs of orthogonal dipoles with four equally spaced sound pressure sensors located in the circle. d represents the distance of two adjacent dipoles and dipole 0 is recognized as the reference point. θ is the angle of incidence. It is assumed that the distance between remote sound source and dipole 0 is r. Based on this assumption, sound wave can be seen as plane wave. The output signal of these four base arrays are $p=p_{i},i=0,1,2,3,4$. M is the receiving sensitivity of the pressure-gradient vector hydrophone. k, j denote wave numbers and imaginary number unit, respectively, and then sound pressure p is given by:

(8) $$p=p_{1}+p_{2}+p_{3}+p_{4}=4Mp_{0}\cos(\frac{kd}{2}\cos\theta )\cos(\frac{kd}{2}\sin\theta )$$

differential pressure in X direction $v_{x}$ is given by:

(9) $$p=p_{1}+p_{4}-p_{2}-p_{3}=4jMp_{0}\cos(\frac{kd}{2}\sin\theta )\sin(\frac{kd}{2}\cos\theta )$$

After a series of calculations with approximation and simplification, sound pressure p(t) is given by:

(10) $$p(t)=4MA_{0}\cos(\omega t+\varphi_{0})$$

differential pressure in X direction $v_{x}(t)$ is given by:

(11) $$v_{x}(t)=-\frac{2d\cos\theta MA_{0}}{c}\omega \sin(\omega t+\varphi_{0})$$

where t, ω, $\varphi_{0}$ are the time instant, angular frequency and initial phase respectively. Then complex signal PV is proposed for widely linear filtering, $PV=p+v_{x}$, which is uncircular in most cases, as calculated in Appendix A:

(12) $$PV(k)=A\cos(\omega k\Delta T+\varphi_{0})-jB\sin(\omega k\Delta T+\varphi_{0})$$

The complex signal *PV* which is deduced in Appendix B can be written as:

(13) $$PV(k)=C(k)e^{j(\omega k\Delta T+\varphi_{0})}+D(k)e^{-j(\omega k\Delta T+\varphi_{0})}$$

Then the ACLMS algorithm according to Equation (7) is given by:

(14) $$\begin{array}{ll} \hat{PV}(k+1) & =v(k)h(k)+v^{*}(k)g(k)\\ \;\;e(k) & =PV(k+1)-\hat{PV}(k+1)\\ h(k+1) & =h(k)+ue(k)PV^{*}(k)\\ g(k+1) & =g(k)+ue(k)PV(k) \end{array}$$

From Equation (13) and (14), the estimate $\hat{PV}(k+1)$ becomes:

(15) $$\begin{array}{ll} \hat{PV}(k+1) & =C(k)h(k)e^{j(\omega k\Delta T+\varphi_{0})}+D(k)h(k)e^{-j(\omega k\Delta T+\varphi_{0})}+C^{*}(k)g(k)e^{-j(\omega k\Delta T+\varphi_{0})}+D^{*}(k)g(k)e^{j(\omega k\Delta T+\varphi_{0})}\\ & =[C(k)h(k)+D^{*}(k)g(k)]e^{j(\omega k\Delta T+\varphi_{0})}+[D(k)h(k)+C^{*}(k)g(k)]e^{-j(\omega k\Delta T+\varphi_{0})} \end{array}$$

while from Equation (13), PV(k+1) can be rewritten as:

(16) $$PV(k+1)=C(k+1)e^{j(\omega k\Delta T+\varphi_{0})}e^{j\omega \Delta T}+D(k+1)e^{-j(\omega k\Delta T+\varphi_{0})}e^{-j\omega \Delta T}$$

Observing the conjugate parts within Equations (15) and (16), the term $e^{j\omega \Delta T}$ and $e^{-j\omega \Delta T}$ can be estimated from:

(17) $$e^{j\omega \Delta T}=\frac{C(k)h(k)+D^{*}(k)g(k)}{C(k+1)}$$

(18) $$e^{-j\omega \Delta T}=\frac{D(k)h(k)+C^{*}(k)g(k)}{D(k+1)}$$

In frequency estimation by adaptive filtering algorithms, at two consecutive time instants, C(k+1)≈C(k), D(k+1)≈D(k). Since $D^{*}(k)=D(k)$, $C^{*}(k)=C(k)$, Equations (17) and (18) can be simplified into:

(19) $$e^{j\omega \Delta T}=h(k)+\frac{D(k)}{C(k)}g(k)$$

(20) $$e^{-j\omega \Delta T}=h(k)+\frac{C(k)}{D(k)}g(k)$$

It is assumed $y=e^{j\omega \Delta T}$, $\frac{D(k)}{C(k)}=x$, then Equations (19) and (20) can be expressed as:

(21) y=h(k)+xg(k)

(22) $$y^{*}=h(k)+\frac{g(k)}{x}$$

Since (21)×(22)=1, the following expressions can be found

(23) $$\left[y-h(k)][y^{*}-h(k)]=g^{2}(k\right)$$

Equation (23) can be re-written as Equation (24), and the final frequency of widely linear extension of the standard linear frequency estimation method is given by Equations (25), (29), and (30):

(24) $$\cos(\omega k\Delta T)=\frac{1+h^{2}(k)-g^{2}(k)}{2h(k)}$$

Solution 1:

(25) $$\hat{f}(k)=\frac{1}{2\pi \Delta T}\cos^{-1}\left(\frac{1+h^{2}(k)-g^{2}(k)}{2h(k)}\right)$$

Since $(21)={\left(22\right)}^{*}$, Equations (21) and (22) can be understood as a quadratic equation with one unknown x:

(26) $$x^{2}g(k)+x(h(k)-h^{*}(k))-g^{*}(k)=0$$

Two possible solutions to Equation (26) are shown in Equation (27):

(27) $$x=\frac{-j\tilde{h}(k)\pm \sqrt{{\left|g(k)\right|}^{2}-{\left(\tilde{h}(k)\right)}^{2}}}{g(k)}$$

where actually, $\tilde{h}(k)$ means the imaginary part of h(k). Since the system frequency is far smaller than the sampling frequency, the imaginary part of y is positive, thus excluding one of the solutions:

(28) $$y=\overset{⌢}{h}(k)+j\sqrt{{\left(\tilde{h}(k)\right)}^{2}-{\left|g(k)\right|}^{2}}$$

The system frequency is therefore estimated in the form of Equation (29) or Equation (30) Solution 2:

(29) $$\hat{f}(k)=\frac{1}{2\pi \Delta T}\cos^{-1}(\overset{⌢}{y})$$

Solution 3:

(30) $$\hat{f}(k)=\frac{1}{2\pi \Delta T}\sin^{-1}\left(\tilde{y}\right)$$

Here, $\overset{⌢}{h}(k)$ represents the real part of h(k). The above Equations (25), (29), (30) are all general widely linear extensions of the standard linear frequency estimation methods which can theoretically be used for estimating instantaneous frequency in a vector hydrophone.

### 3.2. Results of Simulations and Experiments

#### 3.2.1. Results of Simulations

**Figure 3:**
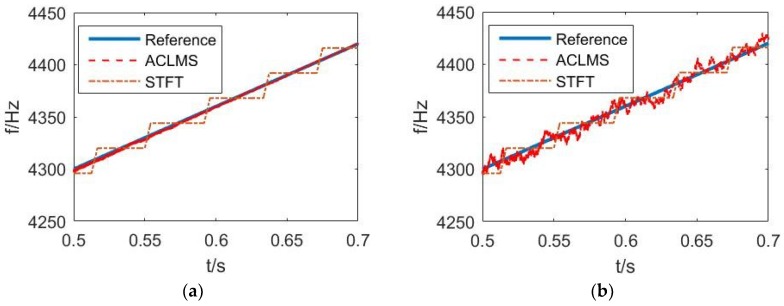
Frequency estimation based on ACLMS and STFT: (**a**) SNR = 57 dB; (**b**) SNR = 37 dB.

The adaptive frequency estimator methods based on the widely linear ACLMS algorithm were applied to estimate the fundamental frequency from sampled values of voltage signals in vector hydrophones, and compared with the standard CLMS algorithm and STFT approach. Simulations were performed in the MATLAB programming environment with a sampling rate of 24 kHz. In the first set of simulations, using Solution 1, Equation (20), the step size μ of both algorithms was set to be 0.0006. It is assumed that the angle of incidence is 30° and all the four sound pressure sensors share the same receiving sensitivity. Firstly, the ACLMS algorithm was compared to the STFT method under different SNR conditions, as is shown in Figure 3.

The LFM signal started from 4000 Hz with modulation rate 600 Hz/s. The step size and window length of the STFT method were set 100 and 1000, respectively. The total signal lasts for one second, but we only observe the stable state from 0.5 s to 0.7 s when it converged. Two dependent WGN sequences were added to the complex simulated voltage signals in the vector hydrophone with different variances. Under rather high SNR conditions (57 dB), the ACLMS algorithm shows smaller estimation variance compared to STFT in Figure 3a, then the SNR was lowered to 37 dB as is shown in Figure 3b. The oscillatory steady-state error of ACLMS rose, while the estimation variance of STFT seems unchanged. To illustrate the statistical advantage of the ACLMS-based estimator over the STFT approach. We calculated the root mean square error (RMSE) of both algorithms in a noisy environment. Table 1 shows the superiority of ACLMS over the STFT algorithm with lower RMSE.

**Figure 4:**
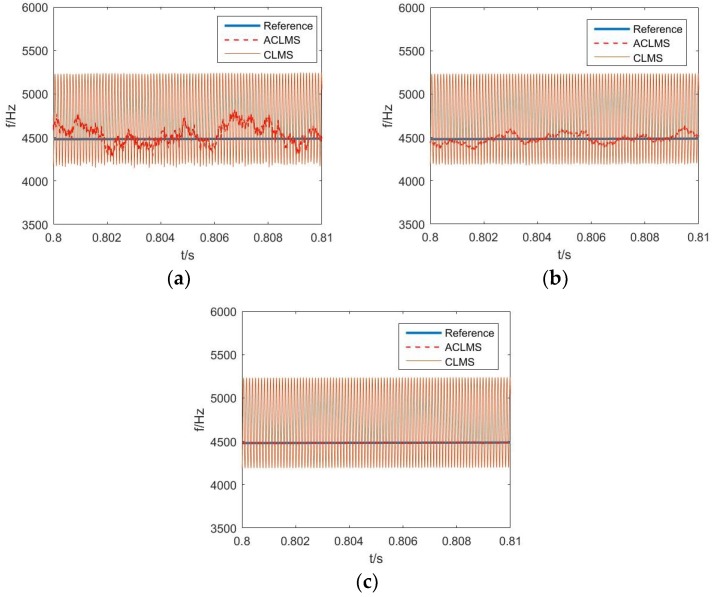
Frequency estimation based on ACLMS and CLMS: (**a**) SNR = 27 dB; (**b**) SNR = 37 dB; (**c**) SNR = 57dB.

In the next simulation, the ACLMS algorithm was compared to the CLMS method. For better observation, a short length of frequency estimation results from 0.8 s to 0.81 s was selected. The total simulation results are illustrated in Figure 4. The advantage of the ACLMS-based estimator over the CLMS-based estimator can be clearly seen under all three different SNR conditions. It is especially noticeable that the estimation error of the ACLMS algorithm decreased with the improvement of SNR, whereas the counterpart of the CLMS algorithm shows no such sign with a stable oscillatory steady-state error.

**Figure 5:**
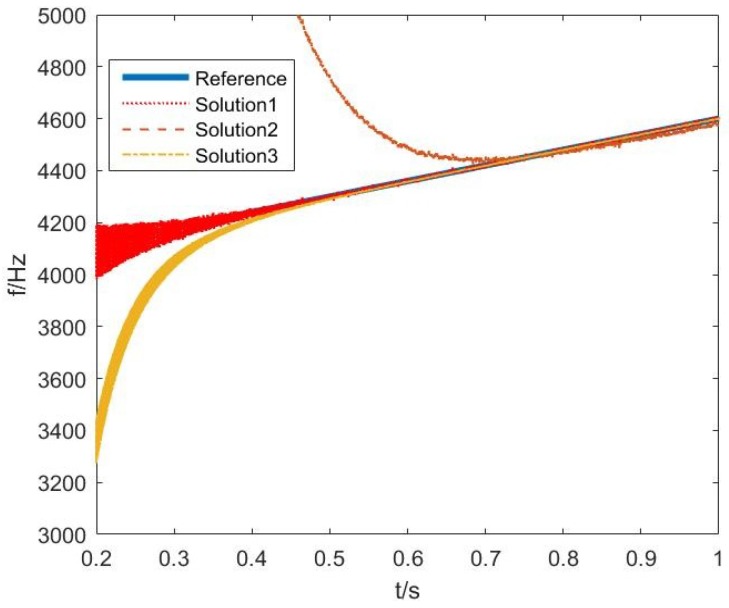
Comparison of convergence rate of three solutions based on ACLMS.

The following set of simulations focused on the performance of three different solutions to the frequency estimation based on the ACLMS algorithm. All the parameters were set the same as those used in the first set of simulations apart from the SNR, which was 47 dB. The simulation results in Figure 5 describe that the convergence rates of the three different solutions are different. Solution 1 converged faster than the others and Solution 2 was the slowest one.

**Figure 6:**
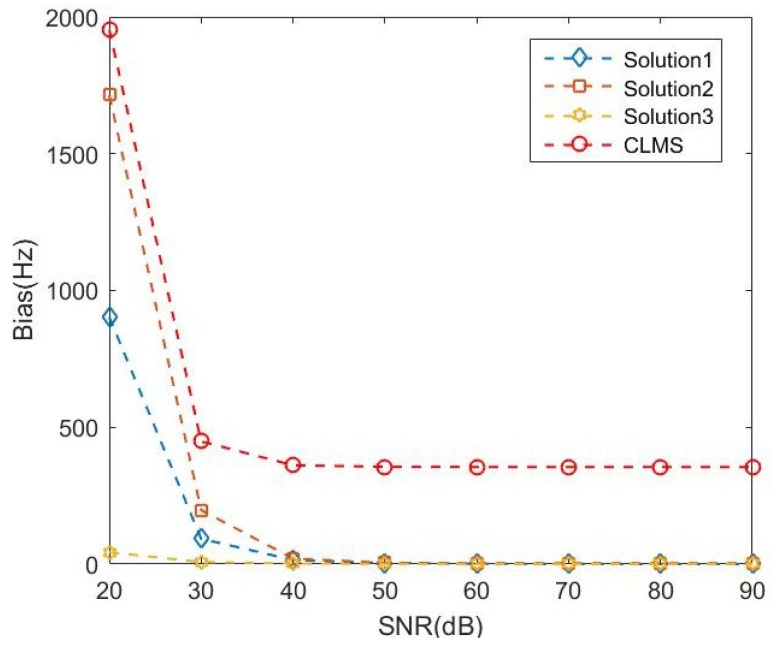
Bias analysis for frequency estimation using three solutions based on ACLMS algorithm and the CLMS algorithm.

The statistical advantage of the widely linear estimators over the corresponding strictly linear estimators is illustrated by comparing the bias in the presence of complex noise at different SNRs. The simulated LFM signal received by a vector hydrophone was assumed to start from 4000 Hz with modulation rate 600 Hz/s lasting for 5 seconds. Statistical analysis only took the latter half frequency results lasting from 2.5 s to 5 s into consideration when the algorithm had converged, which is more meaningful. Figure 6 shows the performance of the three solutions based on the ACLMS algorithm comparing with strictly linear CLMS algorithm at different SNRs. The results in Figure 6 illustrate that three solutions of the ACLMS algorithm and the CLMS algorithm had a decreasing bias as the signal-to-noise ratio (SNR) increased at lower SNRs. The CLMS algorithm yielded relatively larger, almost constant at higher SNRs (bigger than 40 dB). We can observe that the three solutions based on the widely linear algorithm offered the lower estimation error and that, the best performance among them was achieved by Solution 3. The three widely linear model-based solutions generate nearly unbiased frequency estimations at higher SNRs.

#### 3.2.2. Results of Experiments

**Figure 7:**
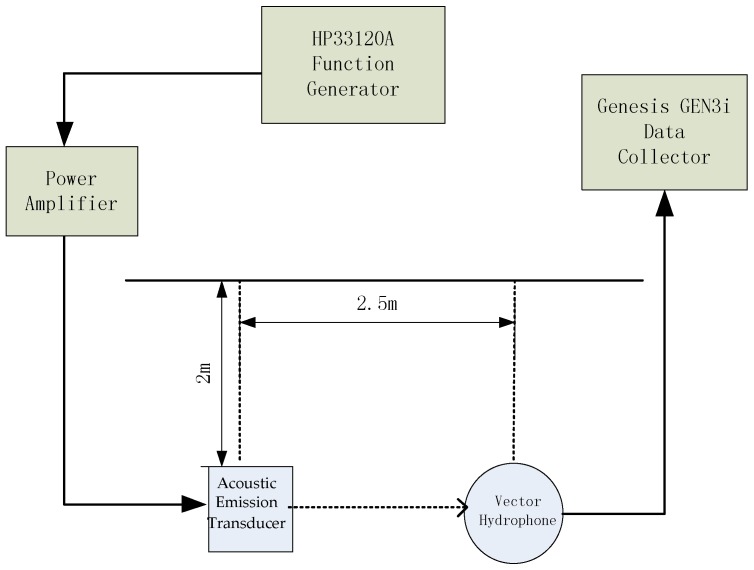
Block diagram of the experiments.

**Figure 8:**
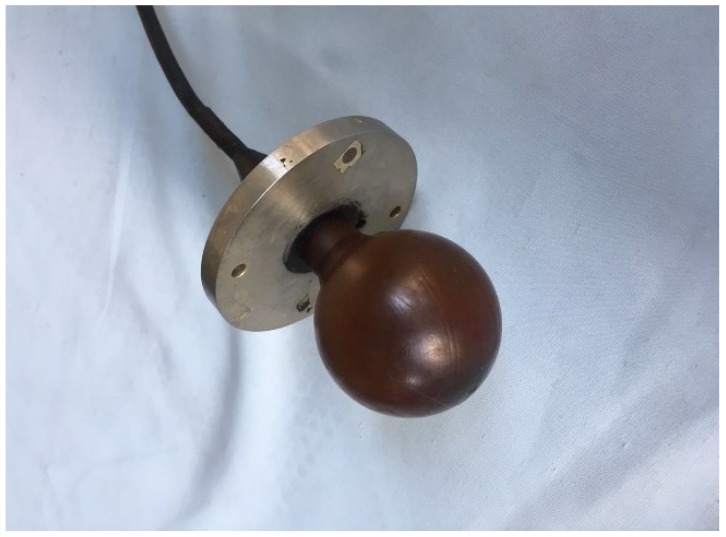
The vector hydrophone.

To support the ACLMS-based frequency estimation approaches and the simulation results above, a series of experiments were conducted in an anechoic tank laboratory. The sound source was put 2 meters below with a horizontal distance 2.5 meters away from the vector hydrophone. The wave files were written in MATLAB and then transmitted to a power amplifier which was connected to the acoustic emission transducer. The flowchart of all the following experiments is shown in Figure 7, and the vector hydrophone used during the experiments is pictured in Figure 8.

**Figure 9:**
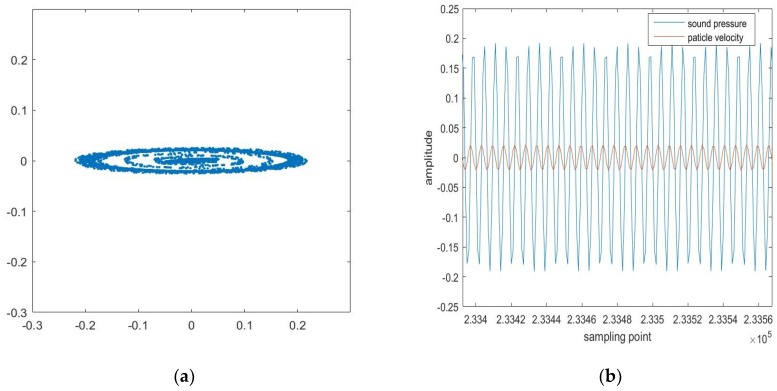
Descriptions of the complex signal PV: (**a**) Scatter diagram via “real-imaginary” plot; (**b**) Time domain wave of the real part and imaginary part of PV respectively.

Firstly, to compare the rate of convergence and the effectivity of the widely linear model-based three solutions to the ACLMS algorithm, a CW signal was transmitted at 4000 Hz level with the duty ratio of one third, and the sampling rate was set at 25 kHz. The data received were then processed in the MATLAB environment. The scatter diagram of the real complex signal PV proposed for widely linear filtering is shown in Figure 9a.

**Figure 10:**
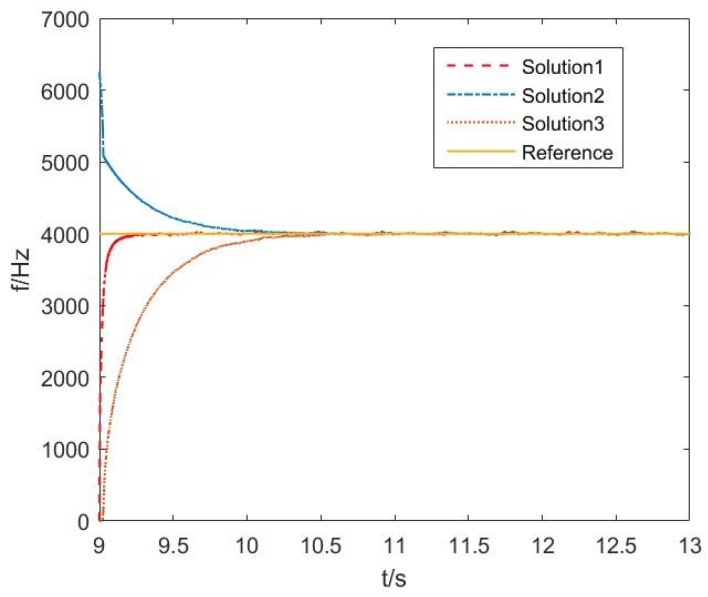
Experiment results of three solutions based on ACLMS algorithm.

Figure 9b gives information about the characteristic that the sound pressure and particle velocity have a 90 degrees phase shift, which is a key characteristic for us to compose complex signals so as to estimate the frequency in vector hydrophones. The circularity index $\eta_{PV}=0.9755\approx 1$. The step size μ was set at 0.3. The data processing result is shown in Figure 10. The convergence rate of the three solutions in real sampled vector hydrophone signals was similar to the simulation results mentioned before, and Solution 1 was also the fastest. It is expected that Solution 1 may be useful for frequency tracking when the system endures the frequency disorders.

**Figure 11:**
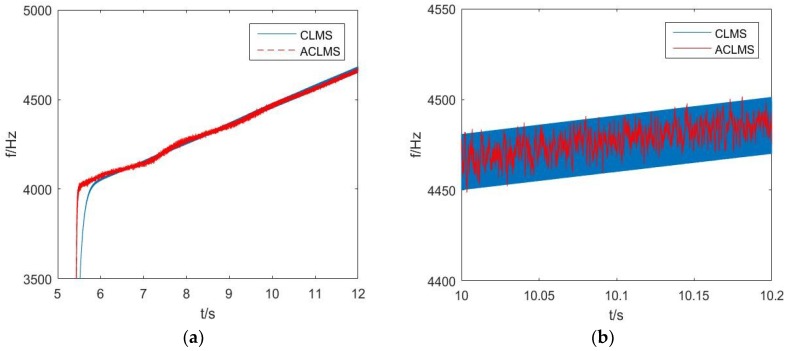
Frequency estimation for a real vector hydrophone system: (**a**) the MATLAB processing result; (**b**) Selected enlarged part of (**a**).

The following experiment considers frequency estimation for the LFM signal received by the vector hydrophone, where the real frequency was initialized at 4000 Hz with the modulation rate 100 Hz/s lasting for 10 s, sampled at a rate of 200 kHz. The MATLAB processing result is shown in Figure 11, where the practical superiority of the algorithms based on the widely linear model, compared with the strictly linear algorithm applied in the frequency estimation in the real vector hydrophone condition is highlighted. In Figure 11a, the convergence rate of the ACLMS-based algorithm is superior to the CLMS-based algorithm. Besides, the oscillatory steady-state error of ALCMS is smaller than that of CLMS as is shown in Figure 11b, which is a selected enlarged part of Figure 11a.

**Figure 12:**
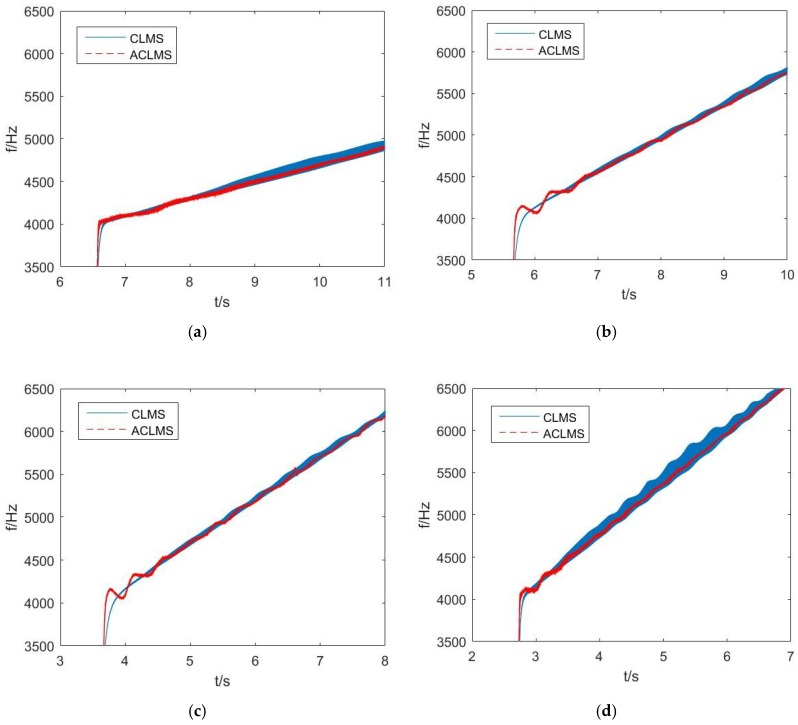
Experiments using LFM signals with different modulation rate: (**a**) 200 Hz/s; (**b**) 400 Hz/s; (**c**) 500 Hz/s; (**d**) 600 Hz/s.

Finally, to test the stability of the algorithm based on the widely linear model and its stable superiority to the strictly linear model, except for the LFM signal with modulation rate 100 Hz/s, other LFM signals with different moderation rate (200, 400, 500 and 600 Hz/s, respectively) were taken into consideration. The SNR is at approximately 40 dB level. Experimental results are shown in Figure 12, and they support the simulation results and the theoretical analysis.

## 4. Conclusions

Theoretically, the sound pressure and particle velocity in a vector hydrophone have a phase difference of 90 degrees, which provides the possibility that a noncircular complex signal can be constructed and it can be transformed into an exponential form and used for widely linear frequency estimation based on recent advances in augmented complex-valued second order statistics. It has been illustrated that the joint variable complex signal PV is second order noncircular, for which the standard linear adaptive CLMS-based estimator is suboptimal. Then a widely linear frequency estimation method of the instantaneous frequency in vector hydrophone has been proposed, which includes the three solutions we concluded and utilizes some recent advances in complex statistics. The three solutions based on the ACLMS algorithm and widely linear model have been shown to be more suitable for signals compared with conventional algorithm based on linear models. The Solution 1 is significant for its fastest convergence rate to normal value. In addition, the ACLMS-based method has the characteristics of faster convergence rate and lower oscillatory steady-state error, and it can reach the unbiased frequency estimation at higher SNR conditions. Future research will focus on dealing with signals at lower SNR conditions, which have rather bigger estimation errors and solving the problem of how to initialize the step size μ at a reasonable value during the MATLAB data processing.

## Figures and Tables

**Table 1:** The value of RMSE of STFT and ACLMS under different SNR conditions.

SNR	STFT	ACLMS
57dB	6.9148	1.9920
37dB	6.9148	5.8826

## Appendix A

The degree of non-circularity ${ϒ}_{PV}$ is calculated using circularity index in Equation (6). Given that:

(A1) $${ϒ}_{PV}=\frac{E[PV^{2}]}{E[{\left|PV\right|}^{2}]}$$

By replacing PV in (A1), ${ϒ}_{PV}$ can be written as (A2), (A2) can also be re-written as (A3):

(A2) $$\begin{array}{ll} {ϒ}_{PV} & =\frac{E[A^{2}\cos^{2}(\omega t+\varphi_{0})]-E[B^{2}\omega^{2}\sin^{2}(\omega t+\varphi_{0})]-E[2AB\omega j\cos(\omega t+\varphi_{0})\sin(\omega t+\varphi_{0})]}{E[A^{2}\cos^{2}(\omega t+\varphi_{0})]+E[B^{2}\omega^{2}\cos^{2}(\omega t+\varphi_{0})]}\\ & =\frac{E[A^{2}\cos^{2}(\omega t+\varphi_{0})]-B^{2}\omega^{2}E[\sin^{2}(\omega t+\varphi_{0})]-2AB\omega jE[\cos(\omega t+\varphi_{0})\sin(\omega t+\varphi_{0})]}{A^{2}E[\cos^{2}(\omega t+\varphi_{0})]+B^{2}\omega^{2}E[\cos^{2}(\omega t+\varphi_{0})]} \end{array}$$

Equation (A2) can be simplified into Equation (A3), since $E[\cos(\omega t+\varphi_{0})\sin(\omega t+\varphi_{0})]=0$:

(A3) $${ϒ}_{PV}=\frac{A^{2}-B^{2}\omega^{2}}{A^{2}+B^{2}\omega^{2}}$$

where $A=4MA_{0}$, using $B=\frac{dA\cos\theta }{2c}$ replace it in Equation (A3) and then:

(A4) $$\begin{array}{ll} {ϒ}_{PV} & =\frac{1-\omega^{2}{\left(\frac{d\cos\theta }{2c}\right)}^{2}}{1+\omega^{2}{\left(\frac{d\cos\theta }{2c}\right)}^{2}}\\ & =\frac{1-\omega^{2}{\left(\frac{d\cos\theta }{2c}\right)}^{2}+2\omega^{2}{\left(\frac{d\cos\theta }{2c}\right)}^{2}-2\omega^{2}{\left(\frac{d\cos\theta }{2c}\right)}^{2}}{1+\omega^{2}{\left(\frac{d\cos\theta }{2c}\right)}^{2}}\\ & =1-\frac{2\omega^{2}{\left(\frac{d\cos\theta }{2c}\right)}^{2}}{1+\omega^{2}{\left(\frac{d\cos\theta }{2c}\right)}^{2}}\\ & =1-\frac{2}{1+{\left(\frac{2c}{\omega d\cos\theta }\right)}^{2}}\\ & \approx 1 \end{array}$$

The velocity of underwater signals is about 1500 m/s, d≈0.01m. The frequency of often used underwater signals is at the kHz level. It can be calculated that ${ϒ}_{PV}\approx 1$ and concluded that complex signal PV is second order noncircular.

## Appendix B

(A5) $$\begin{array}{ll} PV(k) & =A\cos(\omega k\Delta T+\varphi_{0})-jB\sin(\omega k\Delta T+\varphi_{0})\\ & =\frac{A}{2}\left[e^{j(\omega k\Delta T+\varphi_{0})}+e^{-j(\omega k\Delta T+\varphi_{0})}\right]-\frac{B\omega }{2}\left[e^{j(\omega k\Delta T+\varphi_{0})}-e^{-j(\omega k\Delta T+\varphi_{0})}\right]\\ & =(\frac{A}{2}-\frac{B\omega }{2})e^{j(\omega k\Delta T+\varphi_{0})}+(\frac{A}{2}+\frac{B\omega }{2})e^{-j(\omega k\Delta T+\varphi_{0})}\\ & =\frac{A}{2}\left[e^{j(\omega k\Delta T+\varphi_{0})}+e^{-j(\omega k\Delta T+\varphi_{0})}\right]-\frac{B\omega }{2}\left[e^{j(\omega k\Delta T+\varphi_{0})}-e^{-j(\omega k\Delta T+\varphi_{0})}\right]\\ & =(\frac{A}{2}-\frac{B\omega }{2})e^{j(\omega k\Delta T+\varphi_{0})}+(\frac{A}{2}+\frac{B\omega }{2})e^{-j(\omega k\Delta T+\varphi_{0})}\\ & =C(k)e^{j(\omega k\Delta T+\varphi_{0})}+D(k)e^{-j(\omega k\Delta T+\varphi_{0})} \end{array}$$

where $A=4MA_{0}$, $B=\frac{2d\cos\theta MA_{0}}{c}$, $A_{0}$ is a positive constant, $C(k)=(\frac{A}{2}-\frac{B\omega }{2})$, $D(k)=(\frac{A}{2}+\frac{B\omega }{2})$.
